# Functional Food-Derived Urolithins: Molecular Mechanisms, Health Effects, and Interactomics with Proteins and Extracellular Vesicles

**DOI:** 10.3390/molecules31020243

**Published:** 2026-01-11

**Authors:** Nevena Zelenović, Milica Kojadinović, Milica Popović

**Affiliations:** 1 Institute of Chemistry, Technology and Metallurgy, National Institute of the Republic of Serbia, University of Belgrade, Njegoševa 12, 11000 Belgrade, Serbia; nevena.zelenovic@ihtm.bg.ac.rs; 2 Institute of Medical Research, National Institute of the Republic of Serbia, University of Belgrade, Tadeuša Košćuška 1, 11000 Belgrade, Serbia; 3 Faculty of Chemistry, University of Belgrade, Studentski trg 12-16, 11000 Belgrade, Serbia

**Keywords:** urolithins, dietary ellagitannins, bioactive metabolites, nutraceuticals, serum albumin binding, extracellular vesicle, health effect

## Abstract

Over the past decade, research on urolithins has expanded significantly due to their role as mediators between polyphenol-rich diets and human health. Understanding the relationships between ellagitannin intake, gut microbiota composition, and urolithin production is essential for evaluating their biological effects and nutraceutical potential. The primary objective of this review is to critically summarise current knowledge on urolithins, bioactive metabolites derived from ellagitannins in plant-based foods, with a focus on their biosynthesis, bioavailability, protein interactions, and potential therapeutic applications. A comprehensive literature search was conducted using PubMed, Scopus, and Google Scholar to identify studies on urolithin biosynthesis, absorption, transport mechanisms, protein binding, and incorporation into extracellular vesicles. Relevant articles were critically analysed to synthesise current evidence and highlight emerging concepts. Key findings indicate that after absorption, urolithins bind to serum albumin, which facilitates their transport to target tissues, exerting anti-inflammatory and antioxidant actions. Recent evidence also shows that urolithins can be packaged into extracellular vesicles, suggesting novel mechanisms for intracellular transport and potential therapeutic applications. This review highlights gaps in current knowledge and proposes directions for future research to optimise their therapeutic potential.

## 1. Introduction

Urolithins (UROs) represent a class of bioactive metabolites produced in the gut through microbial transformation of phenolic compounds found in various fruits. These metabolites have numerous beneficial effects on human health, including antioxidant, anti-inflammatory, and anti-cancer effects [1]. The significance of UROs research lies in their role as a link between nutrition, microbiota, and metabolism. This connection highlights the relationship between the intake of plant polyphenols and their actual biological effects in the body. UROs are a promising group of bioactive metabolites with broad applications for health maintenance and disease prevention, and further research offers significant clinical potential. Understanding individual differences in their production opens up opportunities for personalised nutrition and the development of targeted therapeutic strategies.

## 2. Methodology

**Figure 1:**
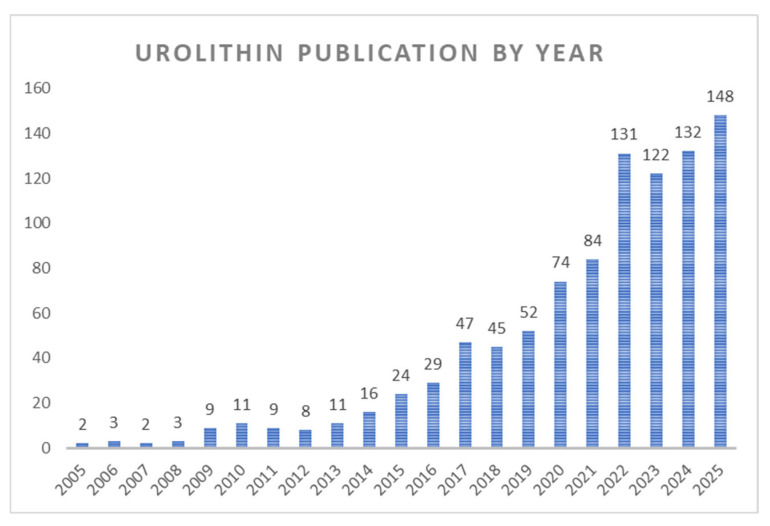
The number of publications related to urolithins has steadily increased over the past decade, with a marked rise after 2020. Data retrieved from PubMed using the search term ‘urolithin’.

This review synthesises current knowledge on urolithin metabolism, molecular mechanisms of action, and systemic effects, integrating evidence from in vitro, animal, and human studies up to 2025. For these reasons, UROs have been the focus of intense research for over a decade. As shown in Figure 1, the number of PubMed-indexed publications related to UROs remained relatively low until 2015, after which a marked increase is observed, reflecting the growing importance of this research field.

This review was based on a literature search designed to collect and critically evaluate current knowledge about UROs derived from functional foods, with particular emphasis on their biosynthesis, bioavailability, molecular mechanisms of action, protein interactions, incorporation into extracellular vesicles, and potential health effects.

The scientific literature for this review was sourced from the following electronic databases: PubMed, Google Scholar, Elsevier, Wiley Online Library, MDPI, ResearchGate and Science direct. In addition to the term urolithin, the following relevant keywords and their combinations were used in the search: ellagitannins (ETs), ellagic acid (EA), intestinal microbiota, bioavailability, protein binding, serum albumin (SA), extracellular vesicles (EVs), mitophagy, antioxidant effect, and aging. Reference lists were manually reviewed, and relevant studies and articles were extracted. Only works that were cited were selected. Articles not written in English, conference abstracts, and studies lacking sufficient experimental or methodological details were excluded.

In addition to manual database searches, an AI-assisted literature exploration tool (tlotoo) was used in selected thematic sections (Section 7, Section 8, Section 9 and Section 10) to support the identification of relevant peer-reviewed publications and emerging research themes. The tool was employed exclusively as a supplementary aid for literature discovery. All references identified through this process were manually verified, critically evaluated, and selected by the authors. No automated data extraction, analysis, or text generation was performed using this tool.

The literature search primarily included studies published in the last 10 years (more than 50% of the publication are from the period 2020–2025). when research on URO expanded. This review also includes earlier studies where necessary to provide historical context. Eligible publications included in vitro, in vivo, and clinical studies, as well as original research articles, relevant review articles and peer-reviewed book chapters that significantly contributed to understanding the metabolism and biological activity of URO. An overview of the literature search, selection, and eligibility assessment is provided in Table 1.

## 3. Biotransformation of Ellagitannins to URO

The positive effects of fruit consumption result from the synergistic interaction of fibre, vitamins, minerals, and polyphenolic compounds found in fruit. Polyphenolic compounds such ETs and EA play an important role in human health (Table 2) [2]. These compounds are present in pomegranate, berries (strawberries and raspberries), nuts (walnuts, pistachios, cashews, and pecans), and grapes. Many of these fruits are considered functional foods, as they provide bioactive compounds that contribute to disease prevention and overall well-being. The main precursors in the human diet include punicalagin and punicalin from pomegranate, casuarictin and pedunculagin from walnuts, and ragrimonin from strawberries.

After consumption of these fruits, ETs are not absorbed in the intestines due to their robust structure. ETs are hydrolysable tannins consisting of a glucose molecule esterified with several units of hexahydroxydiphenic acid (HHDP), which is derived from the oxidative coupling of gallic acid. These complex polyphenols are essentially polymers of gallic acid linked to glucose via ester bonds. Upon hydrolysis, ETs release HHDP, which spontaneously lactonises to EA. EA (2,3,7,8-tetrahydroxy [1]-benzopyranol [5,4,3-cde]benzopyran-5,10-dione; MW = 302 g/mol) occurs in free form, glycosylated and/or acylated, or as ETs polymers [3]. A small amount of free EA can be absorbed directly in the upper gastrointestinal tract. However, ETs are not readily absorbed and are instead partially hydrolysed in the small intestine, releasing more EA [4]. Both ETs and EA have low bioavailability and therefore cannot reach the systemic circulation in significant concentrations. It has been shown that an increase in ET intake does not lead to an increase in the amount of EA in the bloodstream [5]. The unabsorbed EA and remaining ETs enter the large intestine, where they undergo extensive metabolism. Both precursors are metabolised by the intestinal microbiota to 6H-dibenzo[b,d]pyran-6-one derivatives known as urolithins (UROs).

**Figure 2:**
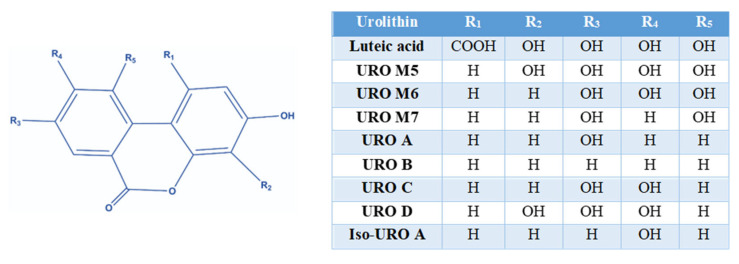
General structures of urolithins and their substitutions.

UROs are a class of organic compounds that contain a benzocoumarin core (Figure 2) but differ in the number and position of hydroxyl groups on the benzocoumarin backbone and in the different R residues depending on the type of URO [6].

**Figure 3:**
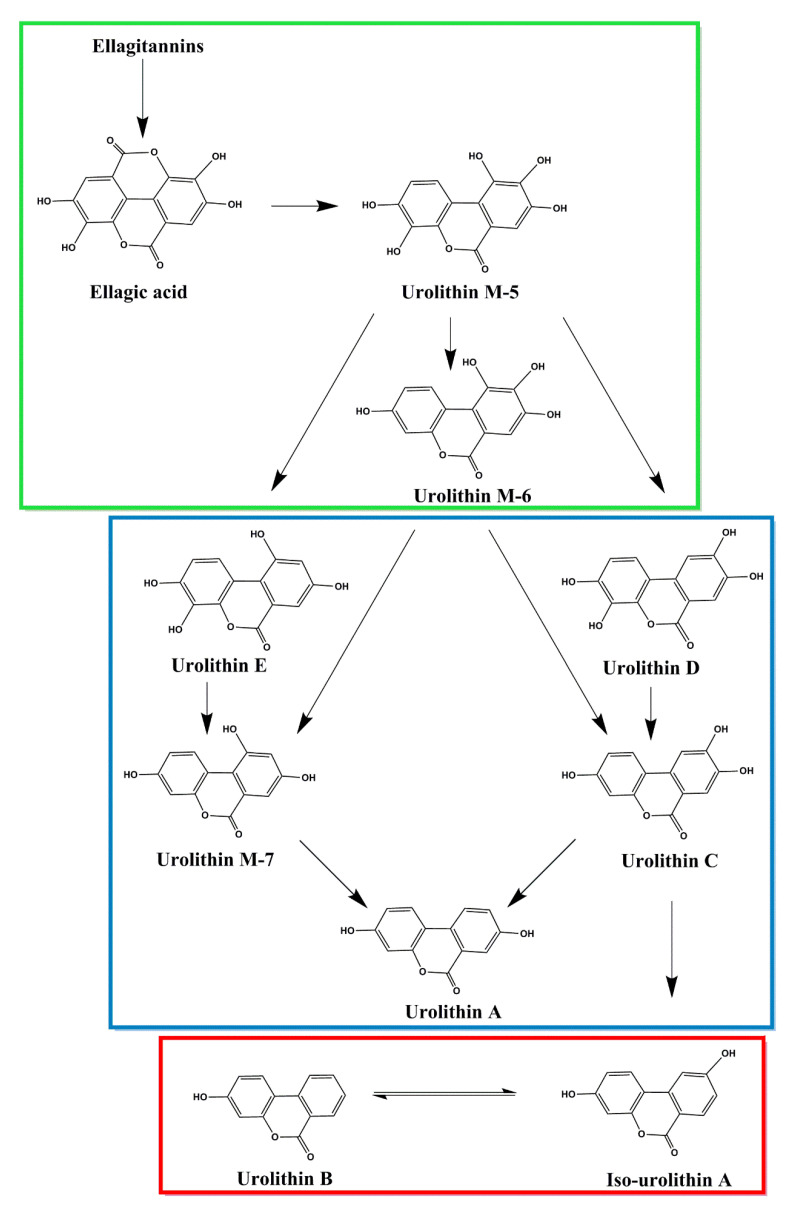
Schematic representation of the degradation pathway of EA and the corresponding urolithin metabotypes: metabotype 0 (green), metabotype A (blue), and metabotype B (red) [9].

The pathway of URO formation begins in the digestive tract when one of the lactone rings of EA is opened and decarboxylated by microbial enzyme activity. After decarboxylation, pentahydroxy-urolithin (URO M5) is formed. This compound [7] is a precursor to various tetrahydroxy-urolithin isomers, which are produced by the removal of a hydroxyl group at different positions (URO D, URO E, and URO M6). Trihydroxy-urolithins (URO C and URO M7) are formed by the removal of the second hydroxyl group, and dihydroxy-urolithins (URO A and iso-URO A) are formed after the removal of a third hydroxyl group. Monohydroxy-urolithin (urolithin B) is formed only after iso-urolithin A [8]. Figure 3 illustrates the degradation pathway of EA and the corresponding URO formation.

Recently, four new UROs were detected in human faeces after the consumption of pomegranates: 4,8,9,10-tetrahydroxy-URO (URO M6R), 4,8,10-trihydroxy-URO (URO M7R), 4,8,9-trihydroxy-URO (URO-R), and 4,8-dihydroxy-URO (URO AR). The production of these new metabolites requires bacterial 3-dehydroxylase activity, which selectively removes hydroxyl groups from the urolithin precursors. This enzymatic activity is attributed to certain anaerobic bacteria in the gut. These bacteria are essential for converting EA into different UROs through a series of dehydroxylation, decarboxylation, and reduction steps [10].

## 4. Role of Gut Microbiota in Urolithin Production

The occurrence and quantity of URO produced in the human body vary greatly between individuals, mainly due to differences in composition of the intestinal microbiota. This inter-individual variability emphasizes the importance of the microbiota as a key factor in converting dietary polyphenols into bioactive metabolites. The biotransformation of ETs is not uniform across individuals, resulting in distinct metabolic phenotypes known as urolithin metabotypes (UMs) [11]. Based on the UROs produced, three different UMs have been identified (Figure 3):

I. Metabotype A (UM-A), characterised by producing only URO A as the final metabolite;
II. Metabotype B (UM-B), characterised by the production of URO B and iso-URO A in addition to URO A;
III. Metabotype 0 (UM-0), in which individuals do not produce any UROs as final metabolites [8,11].

The interaction between polyphenols and the gut microbiome is bidirectional. Firstly, the microbiota influences the production of metabolites and the utilisation of polyphenols; secondly, the individual’s microbial population is modulated. The UM-0 subpopulation can transform into UM-A or UM-B after long-term and/or high exposure to ET or EA [9,12].

The prevalence of UM-A, UM-B, and UM-0 varies in the general population. Among metabolically healthy individuals, UM-A is most common, occurring in about 40–60% of people. UM-B is found in 20–30%, while UM-0, indicating the absence of urolithin metabolites, is found in about 10% of the population. This distribution is not static and can change throughout the lifespan. In children and adolescents, UM-0 is observed more frequently, probably due to an underdeveloped gut microbiota. In adulthood, most people develop UM-A or UM-B, owing to a mature and metabolically active microbiota capable of converting ETs into urolithins. In contrast, in older adults, the prevalence of UM-A tends to decrease, while the proportion of UM-0 increases. This shift is probably a result of reduced microbial diversity and functionality as well as age-related changes in diet, frequent medication use, and general health [13,14].

Numerous bacterial species from the human gut microbiota are involved in the multi-step conversion of EA into UROs. The most important strains of the genus *Gordonibacter* (*G. urolithinfaciens and G. pamelaeae*) produce intermediate UROs (e.g., URO C) but cannot produce either URO A or URO B, as they lack dehydroxylation activity at position 9. However, their presence correlates positively with the presence of URO A in the faeces. *Ellagibacter*, by contrast, can produce iso-URO A via 8-dehydroxylation, but not URO B. It is characteristic of UM-B but can also be present in UM-A individuals, suggesting that additional bacteria are required for the complete conversion pathway. Recent studies have identified species from the genus *Enterocloster* (e.g., *E. bolteae, E. asparagiformis*, and *E. citroniae*) that possess 9- and 10-dehydroxylase activity and can therefore convert URO C to URO A and iso-URO A to URO B—the final steps of the biosynthetic pathway. However, these bacteria cannot metabolise EA themselves and depend on bacteria such as *Gordonibacter*. In co-culture experiments, the combination of *G. urolithinfaciens* and *E. bolteae* resulted in a profile similar to UM-A, while the combination of *Ellagibacter isourolithinifaciens* and *E. bolteae* resulted in a profile similar to UM-B. In addition, new URO metabolites with 3-dehydroxylation (so-called R-UROs) have been identified, whose biological role is still unknown [15].

A key factor in defining UM-A is the presence of bacteria from the genus *Gordonibacter*. The abundance of *Gordonibacter* species in the digestive tract varies with age, health status, and diet, which explains why only about 40% of elderly individuals are able to produce URO A [13]. The URO A produced is more stable and more easily absorbed into the bloodstream than other UROs. This is significant, as URO A is considered the most bioavailable and systemically distributed URO [16].

## 5. Pharmacokinetics, Bioavailability, and Systemic Distribution of URO

Urolithin precursors are poorly absorbed in the gastrointestinal tract:ETs because of their large molecular weight and robust structure, and EA due to its poor solubility in water. Instead, they are microbially degraded in the large intestine, resulting in the formation of UROs. Compared to their dietary precursors, UROs are structurally simpler molecules that are efficiently absorbed and have significantly higher systemic availability. The bioavailability of UROs is greater under intestinal pH conditions due to their lower molecular weight (MW < 300 g/mol), lower polarity, and better stability [6]. Therefore, in 2005, Cerdá and colleagues proposed UROs as the “missing link” to explain the paradoxically low bioavailability of ETs and the reported health benefits of consuming ET-rich foods [11,17].

After being formed in the large intestine by microbial metabolism, UROs are absorbed by the intestinal epithelium and enter the portal circulation. During passage through the liver, they undergo extensive phase II metabolism resulting in conjugation with glucuronic acid or sulfate groups. These conjugated derivatives are the major circulating forms in plasma, while the free (unconjugated) forms are present at much lower concentrations. The concentrations in blood plasma range from 0.003 to 5.2 μM and in urine up to 50 μM. The presence of URO is also characteristic of breast milk from women who consume nuts as an ET-rich food. The total concentration of UROs in breast milk ranges from 8.5 to 176.9 μM, with a predominance of conjugated UROs [11].

Among the conjugated UROs, URO A is the most widely distributed and is mainly present as URO A glucuronide and URO A sulfate, which are detected in human plasma at concentrations between 0.024 and 35 µM. Although these conjugates are more stable and circulate for longer, their exact biological role in vivo remains unclear. In vitro studies suggest that the free form of URO A has greater biological activity than its conjugated counterparts [13].

Clinical studies have shown that URO A appears in human plasma within a few hours after consumption of ET-containing foods and reaches peak concentrations between 6 and 24 h, depending on individual metabolic capacity, which is influenced by the microbiota. The elimination half-life of conjugated UROs ranges from a few hours to over a day, while their excretion via urine can take up to 48 h. UROs have been detected not only in plasma, urine, and breast milk but also in faeces, liver, muscle, and brain tissue [8]. Their detection in the brain suggests that they can cross the blood–brain barrier (BBB), indicating a possible neuroprotective effect. This finding has sparked interest in the use of UROs for the prevention of neurodegenerative diseases [18,19].

## 6. SA as Carriers of URO

The interaction of UROs with biomacromolecules such as proteins is the initial step in biological cascades. Among these proteins, SA plays a key role in transporting UROs in the bloodstream to target tissues, where they perform their biological functions. SA influences the pharmacokinetic fate of UROs, including their half-life in the bloodstream, potency, bioavailability, and tissue distribution. Studying these interactions is important for understanding the pharmacodynamic and pharmacokinetic properties of UROs [20,21].

Human serum albumin (HSA) and bovine serum albumin (BSA) are representative protein models for studying protein–ligand interactions. These proteins exhibit high structural similarity, reflecting their important physiological roles. Both are single-chain, monomeric polypeptides (MW ~ 66.5 kDa) without prosthetic groups. The primary structure of HSA consists of 585, while BSA consists of 583 amino acid residues [22,23]. The secondary structure of these proteins is predominantly α-helical, contributing to their structural stability and flexibility in ligand binding. They have three structurally similar domains (I, II, and III), each organised into two subdomains (A and B) connected by random coil regions [24]. Subdomain IIA contains a binding site referred to as Sudlow I, and subdomain IIIA contains a binding site referred to as Sudlow II [23]. The key structural difference lies in the number and location of tryptophan (Trp) residues contributing to intrinsic fluorescence. HSA has one Trp residue at position 214 (IIA), while BSA has two: Trp 134 (IB, surface-exposed) and Trp 212 (IIA, buried). These distinctions are valuable for spectroscopic analysis of SA-URO interactions [24]. Techniques such as UV/VIS spectrophotometry, fluorescence spectroscopy, synchronous fluorescence spectroscopy, Fourier transform infrared spectroscopy (FTIR), and circular dichroism (CD) spectroscopy, complemented by molecular docking provide sensitive and detailed characterisation of these interactions [25].

Recent studies on SA-URO interactions using these methods have revealed valuable data on their binding affinity, binding sites, and thermodynamic parameters. Fluorescence spectroscopy results show that UROs and their precursor EA quench the intrinsic fluorescence of BSA and HSA without shifting the emission peak, indicating that no major conformational changes occur in the microenvironment of the Trp residue. Each URO binds to a single site on SA with moderate affinity (10^4^ M^−1^) [26,27,28]. Linear Stern–Volmer plots for EA, URO B, URO AG, and URO BG indicate a single static quenching mechanism (Kq ~ 10^12^ M^−1^ s^−1^), while URO A quenches fluorescence by a mixed mechanism involving both static and dynamic quenching [26,27]. Unlike BSA, all investigated UROs quench the fluorescence of HSA by a mixed mechanism. The CD and FT-IR spectra confirm that URO binding does not alter the α-helical structure of HSA [28].

Structural differences among UROs lead to different affinities for the BSA and HSA molecules. URO A has two hydrogen donors in two hydroxyl groups compared to URO B, which has one hydroxyl group and thus one hydrogen donor. Due to this structural difference, URO A can form more hydrogen bonds, resulting in a higher affinity of URO A for BSA [26]. Conjugation of the UROs with glucuronides reduces their affinity for the SA molecule. Free UROs have a higher binding affinity than conjugated UROs, which is reflected in a lower bioavailability in the bloodstream. Molecular docking studies show that URO A and URO B bind favourably to the Sudlow I site of BSA and HSA, while URO C and D prefer the interdomain cavity of HSA. The conjugated UROs bind to the interdomain cavity of SA [26,28].

## 7. URO as Modulators of Cellular Protein Function

**Figure 4:**
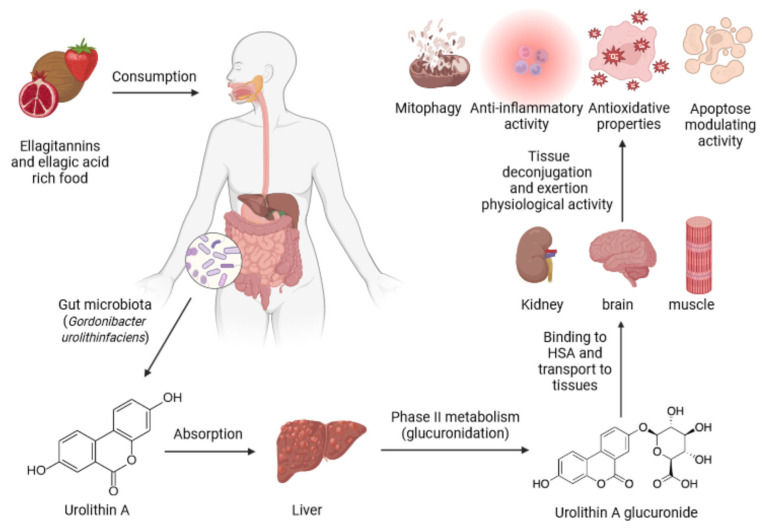
From ET-rich foods to URO A: metabolism, distribution and cellular effects.

As previously emphasised, UROs are transported to peripheral tissues by these proteins where they exert biological activity by interacting with intracellular proteins or enzymes. Through these interactions, UROs mediate a broad spectrum of functions, including anti-inflammatory, antioxidant, anti-proliferative, osteoprotective, and neuroprotective activities [29] (Figure 4). Due to their anti-inflammatory and antioxidant properties, UROs play an important role in the prevention and treatment of chronic diseases associated with inflammation, such as cardiovascular disease, obesity, type 2 diabetes, and metabolic syndrome. The anti-cancer potential of UROs is based on their ability to inhibit cell proliferation, induce apoptosis, and modulate the cell cycle [30]. The most important property of URO A, a representative URO, is its ability to stimulate autophagy in dysfunctional mitochondria [31]. This action is a key mechanism for maintaining cell function and is important in the context of aging and neurodegenerative diseases, such as Parkinson’s and Alzheimer’s [32,33].

*(a)*  *Activation of Mitophagy by Urolithins*

When UROs enter the tissue, they have a beneficial effect on mitochondrial function. UROs stimulate selective autophagy of damaged or dysfunctional mitochondria, a process known as mitophagy, which is a key mechanism for maintaining cell vitality. This process is most often activated by URO A in two ways. The first mechanism involves the PINK1-Parkin-mediated pathway. URO A stabilises the PINK1 kinase, enabling the recruitment of Parkin, which ubiquitinates mitochondrial proteins. PINK1 then phosphorylates the ubiquitin chains, leading to an accumulation of phospho-ubiquitinated mitochondrial proteins. These serve as binding sites for adaptor proteins (optineurin and p62), which then bind LC3 and lead to the formation of the mitophagosome. The second mechanism is the PINK1-Parkin-independent mitophagy pathway. URO A increases the amount of mitochondrial BNIP3 protein, which recruits LC3 directly and independently of Parkin protein. In both pathways, LC3 promotes the formation of a phagosomal membrane around the mitochondria. The resulting mitophagosome fuses with a lysosome. At the end of the process, the low pH in the lysosome and hydrolytic enzymes degrade the mitochondria. Mitophagy increases mitochondrial reserves and is associated with the formation of new organelles, which in turn leads to improved mitochondrial respiratory capacity [16].

Another key mechanism by which URO A regulates mitochondrial quality control is the activation of AMP-activated protein kinase (AMPK), an important cellular energy sensor activated when the cellular ADP/ATP ratio is disturbed. Once AMPK is activated, it restores energy balance by stimulating catabolic pathways that generate ATP, such as fatty acid oxidation, while inhibiting energy-consuming anabolic processes. Simultaneously, AMPK promotes glucose uptake and improves glycogen storage, contributing to metabolic flexibility. Activation of AMPK by URO A not only promotes mitophagy and the removal of damaged mitochondria, but also stimulates pathways that increase the number and efficiency of mitochondria. Importantly, AMPK directly activates peroxisome proliferator-activated receptor-gamma coactivator-1-alpha (PGC-1α), a master regulator of mitochondrial gene expression. Through this AMPK-PGC-1αsignaling pathway, URO A enhances the transcriptional pathways responsible for mitochondrial biogenesis, resulting in increased mitochondrial mass, improved respiratory capacity and more efficient cellular energy metabolism [34].

*(b)*  *Role of Urolithins in Antioxidant Defense Mechanisms*

Mitochondria are organelles present in eukaryotic cells responsible for producing energy in the form of ATP molecules. During this process, electrons are transferred through a complex in the inner mitochondrial membrane, where oxygen acts as the final electron acceptor. Under ideal conditions, oxygen receives four electrons and is converted into water. Some electrons leak from the chain and reduce oxygen, creating reactive oxygen species (ROS) [35]. In small amounts, ROS serve as signalling molecules in mitochondrial biogenesis or adaptation to stress. High levels of these species cause oxidative stress, damage biomolecules, and contribute to aging and diseases [36].

URO A can eliminate ROS directly by acting as a radical scavenger, donating electrons to free radicals such as superoxide anion radicals (O_2_˙ˉ) and peroxyl radicals (ROO^.^), thereby stabilising them and preventing further damage to biomolecules [34]. During oxidative stress, when ROS production increases, URO A can activate Nrf2 (Nuclear Factor Erythroid 2-Related Factor 2), an important regulator of antioxidant defence. Nrf2 is present in the cytoplasm in an inactive form. In the presence of URO A, Nrf2 is released and migrates into the nucleus to trigger the transcription of genes involved in cellular defence [37,38]. Activated Nrf2 binds to the ARE (Antioxidant Response Element) in the promoter regions of genes coding for glutathione S-transferase (GST). This signalling pathway leads to increased expression of GST, an enzyme involved in antioxidant defence. GST enzymes play an important role in maintaining glutathione levels, the most important antioxidant in cells. The increased expression of GST triggered by URO A helps to neutralise oxidative damage [34].

*(c)*  *Urolithins as Modulators of Cell Cycle*

URO A interacts with the Mechanistic Target of Rapamycin (mTOR), a serine/threonine kinase that integrates signals related to cellular energy status and nutrient availability. Activation of the mTOR complex stimulates cell growth and promotes protein and lipid synthesis while suppressing autophagy. Chronic hyperactivation of the mTOR pathway is associated with aging, metabolic disorders and neurodegenerative diseases. URO A counteracts this process by inhibiting mTOR signalling, promoting mitophagy and thus helping to maintain cellular homeostasis and prolong cellular longevity [34].

URO A not only interacts with proteins involved in the regulation of mitophagy and antioxidant defence, but also binds to cyclin-dependent kinases (CDKs), which are important regulators of the cell cycle. Inhibition of CDK activity leads to arrest of proliferation and activation of signalling pathways that promote apoptosis, allowing URO A to prevent uncontrolled cell growth [39]. Urolithin A modulates the nuclear factor kappa-light-chain-enhancer of activated B cells (NF-κB) signalling pathway and thus exerts a strong anti-inflammatory effect. By inhibiting activation of the transcription factor NF-κB and preventing its translocation into the cell nucleus, URO A suppresses the production of pro-inflammatory cytokines such as interleukin-1β (IL-1β), interleukin-6 (IL-6) and tumour necrosis factor-α (TNF-α). The concentration of these cytokines is significantly reduced, leading to attenuation of inflammatory reactions [34].

**Figure 5:**
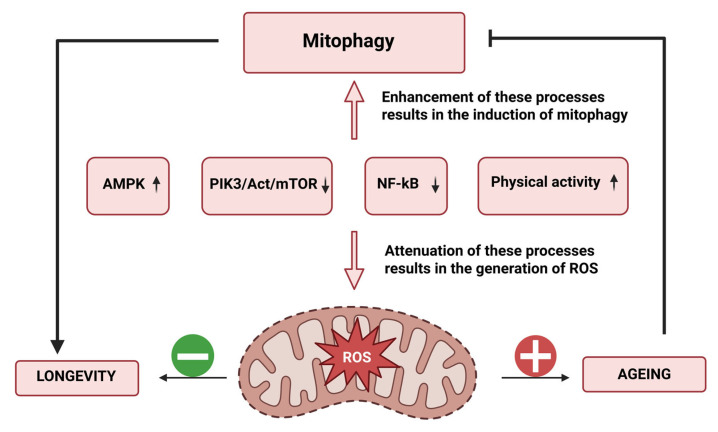
Illustrates the signaling cascades involved in the induction of mitophagy.

Overall, UROs exert their biological activity on two complementary levels. In the bloodstream, their interaction with SA regulates bioavailability, transport, and distribution to peripheral tissues. Once in the cellular environment, UROs interact directly with transcription factors such as AMPK, PGC-1α, NF-κB and mTOR, affecting mitochondrial quality control, antioxidant defence and inflammatory signalling (Figure 5). This highlights the importance of UROs not only as metabolites with favourable pharmacokinetics, but also as active modulators of intracellular signalling pathways critical for cellular homeostasis and health. The development of alternative carrier systems capable of ensuring targeted delivery, while simultaneously protecting UROs from oxidation and conjugation, represents an important future direction to fully exploit their therapeutic potential.

## 8. Biological Effects of URO on Human Health

**Figure 6:**
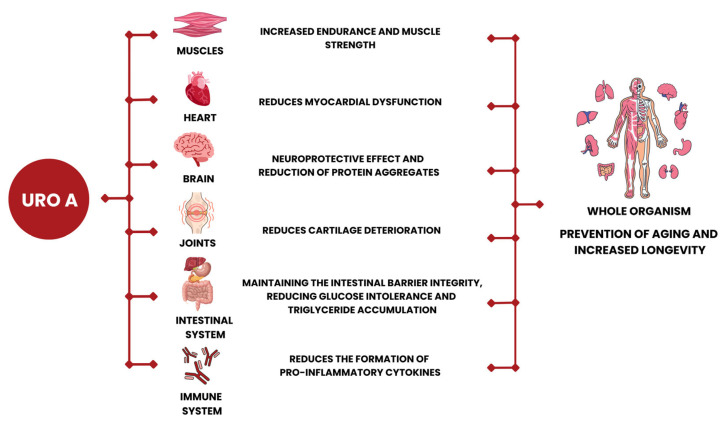
Biological effects of URO A on human health.

Urolithins, especially URO A and URO B, can modulate both physiological and pathological conditions. However, their effectiveness depends on several factors, including the individual’s age, gender, diet, intestinal microbiota composition, and metabolic state. Diet and supplementation can increase urolithin intake. Growing clinical evidence shows that URO A can reshape immunity in healthy individuals and has potential as a systemic therapy against inflammation, oxidative stress, tumours, viruses, and neurodegeneration [14]. Numerous studies provide evidence of its anti-inflammatory, antiapoptotic, and antioxidant properties, as well as its cardioprotective and neuroprotective effects (Figure 6). The beneficial effects of URO A have been observed in various medical conditions such as aging, muscle dysfunction, irritable bowel disease, cardiovascular disease, and metabolic disorders.

*(a)*  *Anti-aging and myoprotective effects of URO A*

One of the first publications to study the direct effects of URO A in vivo examined with their impact on the aging process. Comparing the effects of EA and URO A on the longevity of *C. elegans* roundworms showed that URO A extends their lifespan, while its precursor EA has no effect. URO A also prevents the decline of muscle mass during aging, as indicated by improved muscle fibre integrity and increased mobility in old worms treated with URO A. The positive effect of URO A on muscles during aging has also been demonstrated in mammals, reflected in increased strength and better aerobic endurance [31].

URO A exerts a myoprotective effect on the heart and skeletal muscles, helping to prevent cardiomyopathy and muscular dystrophy by restoring mitochondrial function and enhancing muscle strength and endurance. In models of muscular dystrophy, URO A restores mitochondria in skeletal muscles, increasing their respiratory capacity, strength, and endurance. A decrease in markers of oxidative stress and inflammation is also observed [40].

*(b)*  *Immunomodulatory effect of URO A*

Urolithins modulate key signalling pathways in immune cells. In macrophages, URO A reduces the production of inflammatory molecules known as cytokines and stimulates the production of enzymes involved in antioxidant defence. This balanced activity of URO A involves both suppression of inflammatory processes and enhancement of antioxidative defences in the intestinal tract. URO A, together with its precursor EA and URO B, reduces fibroblast migration and monocyte adhesion, which are key steps in the inflammation process [41,42]. In the immune system, URO A increases the number of lymphocytes and NK cells, enhances CD8+ T cell function, reduces inflammatory cytokines, and boosts cellular energy. These effects strengthen antitumour immunity and open the possibility of combining URO A with immunotherapies, especially in pancreatic cancer, where URO A acts on the PI3K/AKT/mTOR pathway and alters the tumour microenvironment [43,44]. URO A also shows antiviral activity by inhibiting the key enzyme involved in viral replication. Viruses such as herpes simplex virus type 1 (HSV-1) use cyclin-dependent kinase 2 (CK2) for efficient replication. In host cells, the same enzyme activates signalling pathways including NF-κB and STAT3, which stimulate the production of pro-inflammatory cytokines in microglia and neurons. By inhibiting CK2, URO A prevents the replication of HSV-1 and its entry into the nervous system, thereby exhibiting both strong antiviral and anti-inflammatory effects [45].

*(c)*  *Neuroprotective effect of URO A*

In the central nervous system, URO A exhibits neuroprotective properties in animal models of Alzheimer’s disease and multiple sclerosis. Administration of URO A in AD mice improves learning, memory retention, neuronal survival, and neurogenesis in the hippocampus. At the cellular level, long-term URO A treatment in AD models restores autophagy and mitophagy via lysosomal reactivation and reduces Aβ and tau, resulting in functional recovery [32]. URO A shows neuroprotective potential in the brains of people with Alzheimer’s disease by activating mitophagy, reducing inflammation, and decreasing the accumulation of amyloid and tau protein deposits. This mechanism leads to improved cognitive functions in people with Alzheimer’s disease [38]. In mice with MS, administration of URO A reduces dendritic cells, macrophages, and pathogenic Th17 cells, as well as inflammation and demyelination of white matter [46].

*(d)*  *GUT protection by URO A*

In the intestinal tract, URO A showed an anti-inflammatory effect in rats with colitis induced by sodium dextran sulphate (DSS). In the colons of these rats, a decrease in inflammatory markers such as cyclooxygenase-2 protein and a reduction in plasma levels of pro-inflammatory cytokines: interleukin 1 beta (IL-1β), interleukin 6 (IL-6), and tumour necrosis factor alpha (TNFα) were observed [16]. In models of DSS-induced colitis, URO A increases mitophagy markers (NIX, pSer65-PARKIN), improves mitochondrial respiration, and reduces disease severity, linking epithelial mitochondrial quality control to inflammation resolution and barrier integrity during aging [47]. In the gut, URO A helps strengthen the epithelium and restore mitochondrial numbers through mitophagy, which is crucial for treating inflammatory bowel diseases (IBDs). It activates the proteins NIX and PARKIN, improves cellular respiration, and contributes to mucosal recovery, which can be monitored through biomarkers such as CRP and faecal calprotectin [47,48]. URO A is the only known molecule that activates mitophagy, the selective autophagy of damaged mitochondria thereby contributing to mitochondrial renewal and overall cellular health [49].

*(e)*  *Joint protection by URO A*

Aging is a major risk factor for progressive damage to the intervertebral discs of the spine [50]. URO A has been shown to alleviate disc destruction in rats by promoting the production of proteoglycans and collagen, key molecules of the extracellular matrix [51]. URO B has a similar effect in joints affected by osteoarthritis, reflected in reduced inflammation, protection of cartilage, and promotion of autophagy, the cellular cleaning process [52]. Another study shows that protection of discs from damage is associated with increased mitophagy and reduced apoptosis in disc cells [53]. This further supports the role of mitophagy as a key process in various organ systems for maintaining vitality.

## 9. Clinical Implications in Chronic Diseases

Oral administration of UROs has been studied at both preclinical and clinical levels for use as supplements. URO A has recently been generally recognised as safe (GRAS) for use in dietary supplements. Based on this property, a dietary supplement called Mitopure has been approved for human use by the U.S. Food and Drug Administration (FDA). Mitopure provides URO A directly, bypassing the gut microbiome. Clinical studies suggest it plays an important role in preventing diabetes, cardiovascular diseases, and neurodegenerative diseases. Ongoing studies continue to investigate and demonstrate the potential of this molecule [49,54]. Human clinical evidence further supports URO A’s mitochondrial benefits. First-in-human trials confirmed that URO A is safe, bioavailable, and induces a mitochondrial gene signature (including genes for mitochondrial inner membrane complexes) in the vastus lateralis muscle, while lowering plasma acylcarnitines, a marker of inefficient fatty acid oxidation [49].

URO A affects insulin-dependent tissues such as the liver, skeletal muscle, and adipose tissue. When these tissues become resistant to insulin, metabolic syndrome (MetS) develops, accompanied by metabolic dysfunction, reflected in dyslipidaemia, obesity, and glucose intolerance. Supplementation with URO A has a positive effect on these metabolic disorders. Short-term (within two weeks) consumption of pomegranate juice significantly lowered diastolic blood pressure, low-density lipoprotein cholesterol, aminotransferase, and glutathione peroxidase activity in overweight patients aged 40–60 years [55]. Daily intraperitoneal administration of URO A in obese mice reduced hepatic triglyceride accumulation, total cholesterol, and plasma adiponectin levels. Systemic insulin sensitivity is improved. However, no effects on body weight reduction were observed in treated mice [56,57].

Four-month URO A supplementation improves muscle function and mitochondrial health across different age groups. In elderly people (65–90 years), increased resistance to arm and leg fatigue was observed, accompanied by a decrease in C-reactive protein (CRP), a marker of systemic inflammation [58]. Similarly, in middle-aged individuals (40–64 years), URO A supplementation increased leg strength by approximately 10–12%, improved peak VO_2_, and extended six-minute walk distance. These findings indicate that URO A promotes muscle endurance, strength and metabolic efficiency by stimulating mitophagy and reducing oxidative stress, highlighting its potential as a therapeutic intervention for age-related muscle decline and mitochondrial dysfunction [59].

Regarding the immune system and inflammaging (chronic low-grade inflammation associated with aging), a randomised trial in adults aged 45–70 showed that 28-day URO A supplementation was safe and led to reduced systemic inflammation, expansion of lymphocytes and natural killer cells, increased mitochondrial mass in CD8+ T cells, and a shift towards naïve and proliferative phenotypes, indicating improved mitochondrial fitness in immune cells during aging [37]. Mechanistically, URO A directly engages ERK1/2 to activate ULK1 and autophagic flux in CD8+ cells, increasing oxygen consumption rate (OCR), extracellular acidification rate (ECAR), ATP production, and spare respiratory capacity, thereby enhancing antitumour immunity, an immune-metabolic rejuvenation axis relevant to aging [43].

Urolithin A: Neuroprotective, Antitumour, and Cardiometabolic Actions

In neural models, URO A inactivates the TLR3/TRIF signalling pathway, suppresses the NF-κB/STAT1 and ERK/MAPK axes, and upregulates endogenous antioxidant enzymes such as superoxide dismutase (SOD) and catalase. This mechanism effectively reduces innate immune activation that contributes to neuroinflammation [60]. The clinical relevance of this pathway is supported by findings that circulating URO A-glucuronide can be locally deconjugated at sites of inflammation, releasing the pharmacologically active free form of URO A directly into inflamed tissues [42].

Further emphasising its neuroprotective potential, URO A acts as a direct inhibitor of casein kinase 2 (CK2), a key proinflammatory kinase implicated in viral replication and neurodegenerative inflammation. Inhibition of CK2 by URO A and its precursor EA suppresses herpes simplex virus type 1 (HSV-1) replication, reduces viral shedding, and prevents neuroinvasion, thereby mitigating virus-induced brain inflammation [45,60]. Beyond viral models, URO A promotes p62-dependent lysophagy in retinal tissue, facilitating the removal of permeabilised lysosomes and preserving visual function in acute retinal degeneration models. Alongside activation of PINK1/Parkin-mediated mitophagy, these findings illustrate a broader capacity of URO A to sustain proteostasis and mitochondrial integrity in high-energy neural tissues [61,62].

In cancers, URO A exerts multitarget effects by modulating oncogenic signalling pathways and remodelling the tumour microenvironment. In pancreatic ductal adenocarcinoma (PDAC), URO A inhibits the PI3K/AKT/mTOR axis, reduces phosphorylation of AKT and p70S6K, and suppresses tumour growth, accompanied by a decline in myeloid-derived suppressor cells, tumour-associated macrophages, and regulatory T cells. URO A also interferes with PI3K/PDK1 and STAT3 signalling, enhancing apoptosis [44].

Recent clinical data further highlight the immunometabolic dimension of URO A. In a randomised, double-blind trial (MitoIMMUNE), URO A supplementation increased lymphocyte and NK cell counts, enhanced mitochondrial mass and proliferation in CD8^+^ T cells, and reduced circulating proinflammatory cytokines. This remodelling of immune cell energetics and mitochondrial quality control complements its tumour-intrinsic pathway inhibition, suggesting potential for combination immunometabolic therapies [44].

In diabetic cardiomyopathy, URO A improves systolic and diastolic function while suppressing cardiac fibrosis through activation of Drp1-dependent mitophagy. These effects support the principle that mitochondrial dynamism regulates mitophagy in cardiomyocytes, linking mitochondrial turnover to cardiac structural integrity [62,63]. Human trials have confirmed URO A’s safety and its ability to induce a molecular signature of mitophagy in skeletal muscle and blood. In middle-aged and older adults, URO A supplementation enhances muscle strength and endurance while reducing circulating acylcarnitines, consistent with improved mitochondrial fatty acid oxidation and oxidative phosphorylation efficiency. These effects are further supported by recent findings identifying an ETFDH–CIII–COQ2 metabolon that maintains coenzyme Q homeostasis and minimises electron leak, providing a biochemical rationale for the systemic cardiometabolic benefits of URO A [49,59,64].

Moreover, URO A’s suppression of pattern-recognition receptor-driven inflammatory signalling and enhancement of antioxidant defence suggest an additional mechanism for reducing endothelial activation and vascular inflammation in chronic cardiometabolic disorders. Local deconjugation of URO A-glucuronide under inflammatory conditions may further enable pharmacologically active URO A concentrations in affected vascular tissues. Taken together, URO A emerges as a pleiotropic modulator of mitochondrial and cellular quality control with therapeutic potential. By activating mitophagy, lysophagy, and antioxidant defence while attenuating immune overactivation, URO A restores mitochondrial turnover, reduces oxidative stress, and re-establishes metabolic balance, making it a promising candidate for disorders characterised by mitochondrial dysfunction and chronic inflammation [42,60].

Table 3 summarizes the clinical trials investigating urolithin, offering a brief examination of study design, administered dosage, duration, and principal findings. This table serves to elucidate the safety profile, bioavailability, and mitochondrial effects of URO A across various age groups and clinical settings, thereby enabling straightforward comparisons across the trials. Furthermore, it underscores the therapeutic possibilities of URO A in addressing metabolic, muscular, and age-related conditions.

## 10. Interactions with EVs

### 10.1. Influence of UROs on EV Biogenesis and Cargo

EVs, including exosomes and microvesicles, have emerged as critical mediators of intercellular signalling, influencing processes such as immunity, tissue homeostasis, and disease progression. EV biogenesis is tightly linked to cellular stress responses, endolysosomal homeostasis, and cytoskeletal transport, and is regulated by ESCRT-dependent and -independent mechanisms for exosome biogenesis and microvesicle shedding [65]. Urolithins, particularly URO A, regulate mitochondrial quality control, autophagy/mitophagy, and lysosomal activity in a number of tissues, processes that are mechanistically proximal to EV biogenesis and cargo segregation [38,40,66]. In Duchenne muscular dystrophy and Alzheimer’s disease models, URO A restores mitophagy and lysosomal function, respectively, and reprogrammes cellular metabolism and inflammatory signalling, suggesting that EV biogenesis and cargo sorting may be indirectly reprogrammed by URO A-mediated normalisation of endolysosomal flux and redox state [38,40]. As EV cargo reflects the physiological state of donor cells, URO A-induced modification of signalling pathways (e.g., NF-κB/MAPK/Akt inhibition in microglia; Sirt1–AMPK–PI3K/AKT/mTOR activation in metabolic tissue) can bias EV cargo towards anti-inflammatory microRNAs, metabolic enzymes, and mitochondrial/lysosomal proteins consistent with a cytoprotective phenotype [66,67,68]. Although direct, cause-and-effect evidence demonstrating UROs reprogram EV cargo remains limited, these pathway crossovers create firm testable predictions that URO A may suppress pro-inflammatory EV signalling and augment EVs with markers of mitochondrial regeneration and lysosomal performance [38,65,66].

### 10.2. EV-Mediated Delivery of Urolithins and Their Metabolites

EVs are increasingly used as delivery carriers due to their stability, tropicity, and capacity to carry small molecules and biomacromolecules [69,70]. URO A and UROs derived from URO A circulate mainly as phase II conjugates, undergo enterohepatic recirculation, and selectively accumulate in certain tissues (e.g., prostate, colon, intestine) after food intake, demonstrating intricate transport and distribution dynamics [71,72]. Although definitive evidence that UROs are packaged into endogenous EVs is yet to be shown, EVs can package metabolites and influence their pharmacokinetics, and engineered EVs are actively being developed as precision delivery vehicles for small-molecule cargos in various diseases [65,69,70]. Due to URO A’s conjugatability and low free-plasma levels, EV-based formulation—native or designer—could theoretically protect UROs from premature clearance, facilitate tissue targeting (e.g., muscle, brain), and enable combination therapeutics (e.g., co-loading URO A with proteasome inhibitors in cancer) [69,70,73]. These proposals are worth experimental programmes to determine URO quantification in EV fractions and to assess loading efficiencies and bioactivity in engineered EV platforms [69,70].

### 10.3. Role of EVs in Systemic Distribution and Cellular Communication

**Figure 7:**
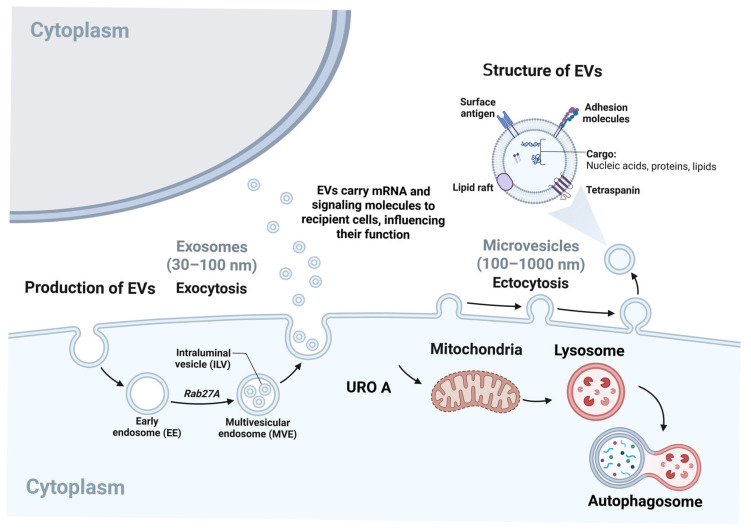
Schematic overview of the interplay between urolithin A, mitochondrial quality control, and extracellular vesicle (EV) biogenesis and signaling.

EVs are systemic messengers that traverse biological barriers and coordinate intercellular communication within immune, metabolic, and neural networks (Figure 7) [69]. URO A’s pleiotropic effects—augmented muscle function via induction of mitophagy, suppression of neuroinflammation and restoration of lysosomal function, and antineoplastic activity against multiple myeloma—involve distributed signalling, which is augmented or relayed by EVs originating from gut epithelial, immune, or metabolically active tissues [38,40,67,73]. In URO A’s conceptual approach, these EVs can (i) recalibrate donor cells towards mitochondrial health and an anti-inflammatory state; (ii) modulate the EV secretome in both quantity and quality; and (iii) exploit EV trafficking to broadcast stress-resolution signals systemically, potentially reaching privileged sites such as the CNS, where EV-mediated communication is increasingly being appreciated [69]. Although the field currently lacks direct monitoring of URO A-related signals in EVs, incorporating EV profiling into established URO A trials and preclinical models could reveal EV signatures predictive of clinical outcomes [58,69].

## 11. Integrative Perspectives and Emerging Therapeutic Potential

### 11.1. Systems Biology Approaches to Urolithin Action

Urolithin biology spans the microbiome–host interface, phase II metabolism, enterohepatic circulation, and multi-organ crosstalk, making it well suited to systems-level investigation. An integrative pipeline could include:

- Multi-omics in responders versus non-responders: parallel metabolomics (URO conjugates), proteomics (mitochondrial/lysosomal modules), and small RNA/EV cargo profiling under URO A exposure in target tissues (muscle, brain, immune cells) [38,58,65,66].
- Network modelling of signalling axes: inference frameworks connecting Sirt1–AMPK–mTOR mitophagy nodes with NF-κB/MAPK/Akt inflammation and lysosomal cathepsin activity to predict emergent phenotypes and identify combination targets (e.g., proteasome inhibition in MM, lifestyle synergy with exercise) [58,66,67,73].
- EV-aware pharmacokinetic/pharmacodynamic (PK/PD) models: explicit compartments for conjugation, enterohepatic recycling, tissue accumulation, and EV exchange to explain low free-plasma URO A but potent tissue-level effects and long-term responses [69,70,71,72].

Such systems strategies are necessary to reconcile heterogeneity in URO production, distribution, and phenotypic actions in the clinic [66,68].

### 11.2. Future Directions in Personalised Nutrition and Therapeutics

Individualisation of urolithin-containing therapies will require the alignment of dietary precursors, microbial capacity, and delivery science to overcome interindividual variability in metabolite production, conjugation, and tissue exposure [72,74,75]. Microbiome stratification by URO metabotype (UM-A, UM-B, UM-0) and remodelling with proven consortia can reprogramme URO A non-producers to become stable producers in vivo, offering a route to standardise effects and enable precision nutrition [76,77]. Recently identified Enterocloster-encoded urolithin dehydroxylases and the ucd operon provide mechanistic levers to engineer or monitor URO A production capacity directly in the gut microbiota [78]. Efficacy may be context dependent; for example, EA impacted non-alcoholic fatty liver disease (NAFLD) phenotypes preferentially better in high-URO A-producing mice and reconfigured the gut microbiota towards increased Akkermansia, highlighting the value of pre-intervention metabotype profiling [18]. In parallel, EV-mediated formulations can deliver and stabilise urolithins to inaccessible tissues, complementing conventional supplementation and probiotic therapies while providing diagnostic information through EV cargo readouts during drug development [69,71]. In aggregate, microbiome-stratified strategies and EV precision delivery may convert UROs from potential nutraceuticals into drugs with predictable and quantifiable, mechanistic biomarkers of response [69,76,78].

## 12. Conclusions

Urolithins are key metabolites that link a polyphenol-rich diet to its beneficial health effects. Consumption of foods high in ETs has shown that the gut microbiota metabolises these compounds into bioactive metabolites, which demonstrate potential in both physiological and pathological conditions. After their formation in the intestinal tract, urolithins undergo phase II metabolism, during which they are conjugated before entering the systemic circulation.

In the systemic circulation, UROs are predominantly present as glucuronide and sulfate conjugates in the low micromolar range, which confers metabolic stability and prolongs their half-life. The ability of UROs to reach peripheral tissues such as the liver, skeletal muscle, and even the brain makes them promising candidates for therapeutic investigation. However, the presence of free forms (aglycones) in certain tissues is of particular interest, as these are thought to have the most potent biological activities, including anti-inflammatory, anti-proliferative, and mitophagy-inducing effects. Their pharmacokinetic behaviour depends not only on the composition of the individual gut microbiota but also on the chemical form in which they circulate—mainly as phase II conjugates. These pharmacokinetic and biological properties have led to growing scientific interest in UROs as biomarkers for ETs uptake and as promising candidates for the development of nutraceuticals and therapeutics, particularly in the areas of healthy aging, cardiovascular protection, and chronic disease prevention.

The fact that only some individuals are able to produce certain UROs, particularly URO A, in biologically significant amounts indicates possible limitations and variability in response to ETs-based nutritional and therapeutic interventions. Understanding the factors that determine the expression of the metabotype is therefore crucial for the development of personalised approaches in nutrition and health. Emphasis has been placed on the production of URO-based supplements, which overcomes dependence on certain strains of the microbiota. For people with low URO production, this approach enables better utilisation of its health benefits.

In recent years, extracellular vesicles have become the subject of intense research as natural nanocarriers due to their origin, ability to cross biological barriers—including the blood–brain barrier—low immunogenicity, and small size. The potential of EVs as carriers for polyphenols opens the possibility for the development of new therapeutic platforms for precise and targeted delivery of bioactive compounds. In addition, EVs protect biologically active compounds from enzymatic degradation, oxidation, or conjugation allowing these compounds to be transported in active form to the site of action.

## Figures and Tables

**Table 1:** Methodology of literature search and study selection.

Step		Description
Identification	Databases	PubMed, Google Scholar, Elsevier, Wiley Online Library, MDPI, ResearchGate and Science Direct
Selection	Keywords	“urolithins”, “ellagitannins”, “gut microbiota”, “bioavailability”, “health effects”
	Search period	2005–2025
	Language	English
	Study types	In vitro, in vivo, clinical studies,
	Article types	Original research articles, review articles
Exclusion	Criteria	Articles not available in full text, conference abstracts, non-English publications

**Table 2:** Fruits as dietary sources of EA (mg per 100 g fresh weight) [2].

Fruit	Major ETs Identified	EA (mg/100 g Fresh Weight)
Pomegranate (*Punica granatum*)	Punicalagin	2.06
Raspberry (*Rubus idaeus*)	Sanguiin H-6, Lambertianin C	1.14
Blackberry (*Rubus fruticosus*)	Lambertianin C, Sanguiin H-6, Pedunculagin, Casuarictin, Castalagin/Vescalagin, Lambertianin D	43.67
Strawberry (*Fragaria × ananassa*)	Agrimoniin, Sanguiin H-6, Casuarictin	1.24
Walnut (*Juglans regia*)	Pedunculagin	28.50
Muscadine grape (*Vitis rotundifolia*)	Specific names not always clearly defined	0.90

**Table 3:** Summary of clinical trial investigating UROs.

Study	Population/Species	Intervention	Duration	Key Findings	References
First-in-human trial	Healthy adults	URO A	Single/multiple doses	Safe, bioavailable; induces mitochondrial gene signature in skeletal muscle; lowers plasma acylcarnitines	[49]
Randomized trial	Adults 45–70 years	URO A	28 days	Reduced systemic inflammation; increased NK cells and mitochondrial mass in CD8^+^ T cells	[37,43]
Middle-aged individuals	Adults 40–64 years	URO A	4 months	Increased leg strength 10–12%, improved peak VO_2_, increased 6 min walk distance	[59]
Elderly individuals	Adults 65–90 years	URO A	4 months	Improved resistance to arm and leg fatigue; decreased C-reactive protein (CRP)	[58]
Overweight adults	Adults 40–60 years	Pomegranate juice (URO precursor)	2 weeks	Lowered diastolic BP, LDL cholesterol, aminotransferase, glutathione peroxidase activity	[55]
Obese mice	Mice	Intraperitoneal URO A	Short-term	Reduced hepatic triglycerides, total cholesterol, improved insulin sensitivity; no body weight change	[56,57]
Neural models	Cell lines RAW264.7/male rats	URO A	Variable	Inactivates TLR3/TRIF, suppresses NF-κB/STAT1 and ERK/MAPK; upregulates antioxidant enzymes; reduces neuroinflammation	[42,60]
Pancreatic ductal adenocarcinoma	PDAC cell lines/mice	URO A	Variable	Inhibits PI3K/AKT/mTOR, reduces tumor growth, modulates tumor microenvironment, enhances apoptosis	[44]
Cardiomyopathy models	Human/animal	URO A	Variable	Improves systolic and diastolic function; reduces cardiac fibrosis via Drp1-dependent mitophagy	[62,63]

## Data Availability

No new data were created or analyzed in this study.

## Abbreviations

The following abbreviations are used in this manuscript:

UROs	Urolithins
ETs	Ellagitannins
EA	Ellagic acid
UM	Urolithin metabotype
SA	Serum albumin
BSA	Bovine serum albumin
HSA	Human serum albumin
EV	Extracellular vesicles
